# “The Problem Is that We Hear a Bit of Everything…”: A Qualitative Systematic Review of Factors Associated with Alcohol Use, Reduction, and Abstinence in Pregnancy

**DOI:** 10.3390/ijerph18073445

**Published:** 2021-03-26

**Authors:** Vivian Lyall, Lindsay Wolfson, Natasha Reid, Nancy Poole, Karen M. Moritz, Sonya Egert, Annette J. Browne, Deborah A. Askew

**Affiliations:** 1 Primary Care Clinical Unit, University of Queensland, Brisbane, QLD 4006, Australia; v.lyall@uq.edu.au (V.L.); d.askew@uq.edu.au (D.A.A.); 2 Centre of Excellence for Women’s Health, Vancouver, BC V6H 3N1, Canada; npoole@cw.bc.ca; 3 Canada Fetal Alcohol Spectrum Disorder Research Network, Vancouver, BC V5R OA4, Canada; 4 Child Health Research Centre, University of Queensland, Brisbane, QLD 4101, Australia; n.reid1@uq.edu.au (N.R.); k.moritz1@uq.edu.au (K.M.M.); 5 School of Biomedical Sciences, The University of Queensland, St. Lucia, QLD 4072, Australia; 6 Southern Queensland Centre of Excellence in Aboriginal and Torres Strait Islander Primary Health Care, Inala, QLD 4077, Australia; Sonya.Egert@health.qld.gov.au; 7 School of Nursing, University of British Columbia, Vancouver, BC V6T 1Z4, Canada; annette.browne@ubc.ca

**Keywords:** Fetal Alcohol Spectrum Disorder, prevention, harm reduction, stigma, trauma-informed, women-centered, qualitative synthesis, women’s health, maternal health, substance use

## Abstract

Understanding the factors that contribute to women’s alcohol use in pregnancy is critical to supporting women’s health and wellness and preventing Fetal Alcohol Spectrum Disorder. A systematic review of qualitative studies involving pregnant and recently postpartum women was undertaken to understand the barriers and facilitators that influence alcohol use in pregnancy (PROSPERO: CRD42018098831). Twenty-seven (*n* = 27) articles were identified through EMBASE, CINAHL, PsycINFO, PubMed and Web of Science. The included articles were thematically analyzed using NVivo12. The analysis was informed by Canada’s Action Framework for Building an Inclusive Health System to articulate the ways in which stigma and related barriers are enacted at the individual, interpersonal, institutional and population levels. Five themes impacting women’s alcohol use, abstention and reduction were identified: (1) social relationships and norms; (2) stigma; (3) trauma and other stressors; (4) alcohol information and messaging; and (5) access to trusted equitable care and essential resources. The impact of structural and systemic factors on prenatal alcohol use was largely absent in the included studies, instead focusing on individual choice. This silence risks perpetuating stigma and highlights the criticality of addressing intersecting structural and systemic factors in supporting maternal and fetal health.

## 1. Introduction

Globally, an estimated 10% of women consume alcohol during pregnancy, with the highest rates of alcohol use during pregnancy being found in Russia (36.5%), the United Kingdom (41.3%), Denmark (45.8%), Belarus (46.6%), and Ireland (60.4%) [1]. Despite ongoing public health efforts to address alcohol use during pregnancy in countries like Australia, Canada, Denmark, France, and the USA, rates are expected to increase [1,2,3].

In Canada and the USA, researchers have noted that while the prevalence of alcohol use remains higher among boys and men, the gender gap is narrowing, particularly between young adults. In some countries, this convergence has been attributed to changes in gender norms and roles [3,4]. Greater economic power, women’s entry into the workforce, and more equitable cultural and economic circumstances may be some reasons for an increased parity of alcohol use rates between men and women [3]. However, an increase in products and targeted advertising towards women that posits alcohol as fun—increasing social connectedness, friendships, and sexual attraction, and as an aide for coping and relaxation, [5,6,7] are also likely factors informing women’s increasing alcohol use.

The increase in women’s alcohol use is cause for concern. Researchers have identified sex-specific effects of alcohol on health, which have prompted the release of national guidelines related to low and lower risk alcohol use [8,9,10]. Moreover, increased rates of alcohol use during pregnancy remains a serious public health concern, as prenatal alcohol exposure (PAE) can lead to miscarriage, stillbirth, premature birth, or result in Fetal Alcohol Spectrum Disorder (FASD), a disability and diagnostic term which refers to the lifelong brain- and body-related impacts of PAE [11].

Understanding the factors that contribute to women’s alcohol use in pregnancy is of critical importance to FASD prevention. Given the normalized role of alcohol use in daily life and social occasions in many societies, it is not uncommon for women to unknowingly consume alcohol prior to pregnancy recognition [12,13,14]. However, for those who use alcohol post-pregnancy recognition, intersecting contextual factors may influence their use, such as: peer influences and social pressures; limited provision of prenatal alcohol use risk information due to discomfort on part of health and social care providers to discuss alcohol use with women and their support networks; or conflicting or unclear information received from health care providers surrounding ‘safe levels’ of alcohol during pregnancy [15,16]. Confusion around what is safe may also result from women’s exposure to conflicting messaging in public discourse, or from family and friends, the media, or online pregnancy content where information around healthy behaviours during pregnancy may be outdated, incorrect, or not evidence based [17,18].

Alcohol use in pregnancy may also be influenced by a range of contextual and structural factors, including poverty, histories of trauma and violence, physical and mental health concerns, sociocultural and economic vulnerabilities and disadvantage, and child welfare involvement [19]. Pregnant and parenting women who use substances such as alcohol can often face a number of personal, institutional, and systemic barriers to accessing services. These include discrimination, racism, stigmatization, lack of mental health support, and avoidance of health and social services out of fear of punitive responses [19].

Despite these challenges, pregnancy is a period of transition that can represent changes in women’s personal identity, daily life, responsibilities, and relationships [19,20]. Parenting can place additional demands and stress on women, impacting the wellbeing of new mothers [20]. This is often exacerbated among Indigenous women, women of colour, and women of a lower socioeconomic status (SES), where there has been oversurveillance, ongoing stigma, and a lack of meaningful attention to the impacts of colonization and intergenerational trauma on individuals and communities [21,22].

There is a small but growing body of qualitative literature that explores women’s perspectives, and privileges their voices, in the discourse relating to alcohol use, abstention, and reduction during pregnancy. This systematic review undertook a detailed analysis of the available literature that explicitly used qualitative research methods to understand the contexts, conditions, and factors influencing women’s use of alcohol during pregnancy. By doing so, the aim of the systematic review was to understand the complexities of women’s alcohol use during pregnancy, advancing what is known about the challenges that women may experience when trying to reduce or abstain from alcohol use during pregnancy, and helping inform and respond to ongoing efforts to support women’s health and prevent FASD.

## 2. Materials and Methods

### 2.1. Protocol and Registration

The study was registered with PROSPERO (CRD42018098831) and reported according to the Preferred Reporting Items for Systematic Reviews and Meta-Analysis (PRISMA) guidelines (see the Table S1 for the PRISMA checklist) [23] and the enhancing transparency in reporting the synthesis of qualitative research (ENTREQ) statement [24]. The systematic review followed the four stages of qualitative synthesis described by Thomas and Harden [25]: (1) searching; (2) quality assessment; (3) data extraction; and, (4) thematic synthesis.

### 2.2. Selection Criteria

#### 2.2.1. Inclusion Criteria

Following the guidance of the ‘SPIDER’ (Sample, Phenomenon of Interest, Design, Evaluation, and Research type) search tool for qualitative studies, primary research articles were included for review if (i) the sample was pregnant and recently postpartum women, (ii) the phenomenon of interest was alcohol use, reduction, or abstinence during pregnancy, (iii) the study design included interviews, focus groups, or participant observation and analysis methods, (iv) women’s views and attitudes were assessed, and (v) the research type was qualitative. For the purpose of our review, recently postpartum was defined as up to three years. Articles that used a mixed methods approach but where qualitative data could be extracted were considered for inclusion. Similarly, studies that met the inclusion criteria, but also involved some non-eligible participants, such as non-pregnant women, women without children, older women, partners, family members, and healthcare professionals were also considered for inclusion. Studies were not excluded based on their epistemological assumptions and/or theoretical traditions.

#### 2.2.2. Exclusion Criteria

Secondary analyses, grey literature, and research published in languages other than English were excluded. Studies where the participants were not identified as pregnant or recently postpartum, where the postnatal period was undefined, or that only included women more than three years postpartum were excluded. Moreover, studies where pregnant or recently postpartum women’s voices were not identifiable (e.g., among women of reproductive age) or where the focus was not primarily on alcohol use in pregnancy (e.g., primary focus on opioid or tobacco use) were also excluded.

### 2.3. Search Strategy

Potential studies for inclusion were identified by conducting a systematic search without any filters, date, or document type restrictions of the following electronic databases: EMBASE; CINAHL, PsycINFO, PubMed, and Web of Science. Additionally, PubMed’s ‘ahead of print’ notifications were used to locate papers yet to be indexed and publication alerts were used to receive notifications of papers published during the review process after formal searches were completed. The following search terms were used: (women OR woman OR maternal OR prenatal* OR pre-natal* OR pregnan* OR primigravida) AND (alcohol* OR fetal alcohol OR foetal alcohol OR alcohol expos* OR alcohol use disorder OR binge OR drink*) AND (qualitative OR grounded theory OR hermeneutic OR thematic OR theme OR phenomenological OR lived experience OR mixed methods). While all of the searches used the same terms, database-specific approaches were applied. See the Supplementary File S1 for the full details of the search strategy.

**Figure 1:**
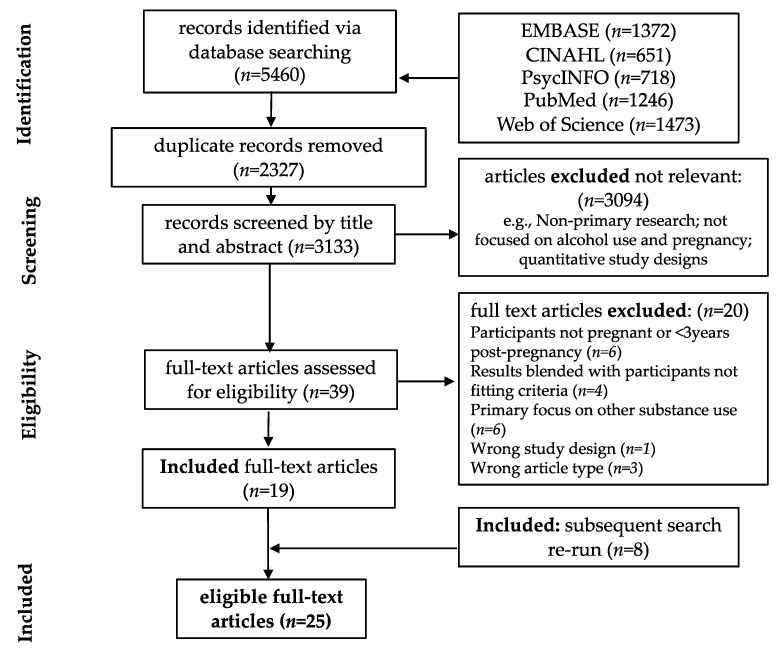
PRISMA Flow Chart.

One author (VL) removed duplicates and screened articles by title and abstracts in accordance with the inclusion criteria. Three authors (VL, NR, and LW) independently reviewed full-text versions of remaining articles. Discrepancies were resolved through a consensus process involving five authors (VL, NR, LW, DA, and KM) (Figure 1).

### 2.4. Quality Appraisal

The consolidated criteria for reporting qualitative research (COREQ) [26] were used to assess the quality of reporting of the included studies. The COREQ contains 32 items that are grouped into three domains: (1) research team and reflexivity; (2) study design; and (3) analysis and findings. As the checklist does not provide a scoring system, we created a scoring system to ensure consistency and transparency in the appraisal process. Here, studies were appraised as either fulfilling (≥30 of the 32 items present), partially fulfilling (≥15 to <30 of the 32 items) or inadequately (<15 items present) fulfilling the criteria. Articles were independently appraised by a combination of two of three authors (LW, VL, DA). Discrepancies in independent assessments were resolved through discussion among the three authors until consensus was reached.

### 2.5. Data Extraction

The following data were extracted from included papers: country; study aims; participants; pregnancy or postpartum status; setting; study design; data collection; analysis approach; and key findings. Data were extracted by two authors (LW and VL) and checked by a third author (DA).

### 2.6. Analysis and Synthesis

To enable transparency, a thematic synthesis approach was selected to make explicit links between the results of each included study and synthesized outcomes [25]. Thomas and Harden [25] outline three stages of thematic synthesis: (1) ‘line-by-line’ coding of text; (2) the development of ‘descriptive themes’; and (3) the generation or application of ‘analytic themes’ which were explored using the *Stigma Action Framework* [27], described below.

To ensure quality, rigor and transparency in the coding process, three conceptually rich papers [28,29,30] were selected as index articles to inform initial development of the line-by-line codebook. These articles were considered conceptually rich due to their comprehensive understanding and coverage of diverse topics, including the impacts of the social and structural determinants of health on alcohol use in pregnancy, from the broader literature on alcohol use during pregnancy and FASD prevention. Index articles were selected by one author (VL) and reviewed by two authors (LW and DA). Using NVivo12, findings specific to women who were pregnant or up to three years postpartum from the results sections of each study were coded according to the contexts, conditions and factors influencing women’s reduction, abstinence or alcohol use during pregnancy. Initial line-by-line coding was conducted by one author (VL) and reviewed by a second author (DA). Following in-depth discussion, a preliminary line-by-line codebook was decided upon, which was then developed iteratively through coding the results sections of the remaining included studies. The remaining coding was done by one author (VL) and reviewed by two authors (DA and LW).

Descriptive themes that closely reflected the core findings identified through line-by-line coding were identified by one author (VL) and reviewed by two authors (DA and LW). The descriptive themes were then discussed in depth by research team members (VL, LW, DA, NP, KM, NR) and different analytical pathways were considered that took into account both established and emerging directions in FASD prevention literature [15,19,21]. The preliminary descriptive coding of findings revealed a strong focus on individual behaviouralist approaches to understanding women’s use of alcohol in pregnancy, with limited attention to the contextual social, and particularly structural determinants, that can influence women’s reduction, abstinence, or continued use of alcohol during pregnancy. To reduce the likelihood of perpetuating stigma, it was decided to apply an analytical framework that had the capacity to bridge the gap between an individualist behavioural lens and the broader substance use, pregnancy, and parenting literature, which includes consideration of the pervasive roles of social and structural determinants in substance use [19,21,31,32].

Consensus was reached that *Stigma Action Framework* was well suited for enabling contextualized understandings of women’s experiences. This framework released by the Canadian Chief Public Health Officer as part of the 2019 report *Addressing Stigma: Towards a More Inclusive Health System*, conceptualizes stigma at the individual, interpersonal, institutional and population levels, allowing for an understanding of how stigma and related barriers and enablers are pervasive at these interconnected levels [27]. In doing so, this framework was also considered pertinent for identifying gaps in the literature limiting comprehensive understandings of factors that may contribute to women’s alcohol use during pregnancy, as well as factors that may support abstinence or reduction. Table 1: Stigma Action Framework has been adapted from the first column of the *Action Framework for Building an Inclusive Health System* to demonstrate how stigma operates at each of the four levels [27].

The *Stigma Action Framework* was adapted using contextual findings from the descriptive coding process and applied to the preliminary results by one author (VL) and reviewed by two authors (LW and DA). Analytical findings were then discussed at length by the research team members (VL, LW, DA, NP, KM, NR, SE), and consensus was reached upon their appropriateness to represent and critique the body of literature. Upon completion of the analytical coding process, themes and findings were critically reviewed by the Indigenous research team member (SE), and feedback was provided regarding the appropriateness and limitations of the qualitative synthesis for Indigenous communities.

## 3. Results—Studies Identified

### 3.1. Study Selection and Characteristics

The initial database search (18 September 2018) identified 5460 articles. Following the removal of 2327 duplicates, 3133 records were screened by title and abstract. Subsequently 39 articles were assessed at the full-text level. Nineteen articles met the inclusion criteria. An updated database search was run prior to publication (4 December 2020) identified eight additional eligible articles, resulting in a total of 27 included articles (Figure 1).

The included 27 articles were published between 1990 and 2020, inclusive (see Table 2: Table of Characteristics). Two articles reported on the same study [33,34] and two articles used the same datasets as previously published research [35,36,37,38]. Of the 24 unique studies, seven were from Australia [29,30,35,39,40,41,42], five from the USA [33,43,44,45,46], two from the UK [47,48], two from South Africa [28,49], two from Switzerland [37,50], two from Brazil [51,52], one from France [53], one from India [54], one from The Netherlands [55], and a joint study from the UK and Sweden [56]. While several studies included partners of pregnant or parenting women, healthcare providers, or community members, this systematic review is only reporting on the pregnant and recently postpartum women who participated. Using this inclusion criteria, studies included 557 participants between the ages of 13 and 45 years.

Six studies focused on women experiencing low SES (*n* = 146 participants) [28,44,46,47,49,54], two reported low-to-medium SES (n = 34 participants) [43,51], and seven reported medium-to-high SES (*n* = 197 participants) [37,42,48,50,52,55,56]. SES was not reported in seven studies (*n* = 157 participants) [29,30,39,40,41,45,53] and was unclear in two studies (*n* = 23 participants) [33,35]. Ethnicity was not reported in 13 studies (*n* = 305 participants) [35,37,39,40,41,44,46,47,48,49,53,55,56]. The remaining 11 studies included participants that identified as Caucasian Australian (*n* = 59) [30,42], Black or Coloured South African (*n* = 24) [28], Caucasian American (*n* = 24) [33,43], Santal or Munda in India (*n* = 19) [54], non-Indigenous Australian (*n* = 15) [29], Aboriginal and Torres Strait Islander in Australia (*n* = 14) [29], Caucasian Brazilian (*n* = 13) [51,52], Indigenous or African American (*n* = 11) [45], Black Brazilian (*n* = 10) [51,52], African American (*n* = 7) [43], Mixed-ethnicity Brazilian (*n* = 4) [51,52], Brazilian Other (*n* = 1) [52], and Asian Australian (*n* = 1) [30].

Qualitative study methods included combinations of interviews (*n* = 21) [28,29,33,34,35,36,40,41,42,43,44,46,47,48,49,50,51,52,54,55,56], focus groups (*n* = 8) [29,30,39,41,45,49,54,55], participant observation (*n* = 2) [33,34], diary entries (*n* = 2) [33,34], online chat room discussions (*n* = 1) [53], and visual data production (*n* = 1) [47]. The majority of studies adopted an individual behaviouralist focus when exploring women’s experiences concerning alcohol use during pregnancy [29,33,34,35,36,37,39,40,41,42,43,45,46,47,48,50,51,52,53,55,56]. This focus highlighted a predominant interest in women’s personal views, values, knowledge and interpretations of information received, behaviours and motivations surrounding alcohol use during pregnancy and, to a lesser extent, with their social and structural contexts. See Table 2 for further details and characteristics from the included literature.

### 3.2. Quality Assessment

Only 7% (2/27) of the included articles fulfilled the COREQ criteria, with 70% (19/27) partially fulfilling and 22% (6/27) inadequately fulfilled the criteria (see Table 2). Articles that only partially fulfilled criteria most commonly did not describe the research team, including interviewer characteristics, credentials, occupation, gender, or relationship with participants within their respective articles. Articles that inadequately fulfilled the criteria similarly excluded descriptions of the research team, as well as information about data saturation, duration of interviews/focus groups, the relationship of participants to the research (such as, if transcripts were returned to participants for comment) and the coding process. All articles, regardless of their quality assessment, provided information about participant selection, setting, the data collection process, and reporting. As such, each contributed conceptually and analytically to the qualitative synthesis.

## 4. Results—Qualitative Synthesis

Five analytical themes emerged from our analysis, which articulated the contexts, conditions and factors influencing women’s reduction or abstinence from alcohol: (1) social relationships and norms; (2) stigma; (3) trauma and other stressors; (4) alcohol information and messaging; and, (5) access to trusted equitable care and essential resources. Each theme explored social and societal impacts on women’s alcohol use and behaviours using the levels of the *Stigma Action Framework* (individual, interpersonal, institutional, and population).

Overall, women’s positionality influenced their relationship with alcohol during pregnancy, including their use, reduction or abstinence from alcohol. Across all articles, women commonly expressed a desire to protect their baby from harm and make positive life changes that facilitated this. However, social and structural determinants of women’s health and wellbeing profoundly mediated their capacity to achieve this at the individual level. Many participants did not disclose experiences of discrimination or serious life stressors (including economic insecurity, unstable or unsafe relationships, or other substance use) [29,35,36,37,39,40,41,42,48,50,53,55,56], however; that does not mean they were not occurring, and for women facing serious challenges such as addiction or multiple life stressors, alcohol reduction or abstinence was not always possible [28,33,34,46,49,51,54]. For those women who reduced or abstained from alcohol in the face of challenging circumstances, the presence of support, whether from a partner, healthcare provider, or through spiritual practice or belief was imperative for enabling women’s personal capacity, including their transition to motherhood and positive attachment to their baby [28,33,34,46,47,49].

### 4.1. Social Relationships and Norms

At the interpersonal and population levels, norms of alcohol abstinence during pregnancy were prevalent in some women’s social environments [29,37,38,41,56]. Abstinence norms constituted important facilitators for some [37,38,56], while for others they created pressure and resulted in stigma and discrimination (see the *Stigma* theme for further details). Alcohol was embedded in most women’s family and social lives across all articles, commonly used in celebrations and for general relaxation.

*“…it [alcohol use] goes along with a social occasion and it goes along with a celebration…”*

[36]

Social norms and environments characterized by low-to-moderate alcohol use were most common [29,30,35,36,37,38,39,40,41,42,43,44,47,48,50,52,53,55,56], with only a minority of women immersed in heavy drinking environments [28,45,49,51,54]. Despite differences in consumption levels across all articles, alcohol use was integral to many women’s social identities, functioning, and relationships. As such, social norms where drinking alcohol was commonplace presented several barriers to reducing or abstaining from alcohol use for women.

*“…Especially if they’re teenagers, all of their friends are teenagers and all of their friends are out drinking. They want to follow their friends and drink”*

[29]

Across moderate-to-heavy drinking contexts [28,33,34,45,46,49,51,54], many women experienced varying degrees of social pressure to consume alcohol from their peer groups, family members, and partners [28,29,30,37,39,43,46,47,49,50,54]. Readily available alcohol combined with a lack of support for some women to reduce or abstain were barriers experienced throughout all pregnancy stages [28,45,46,49,51,54]. For instance, in a study of women in Odisha, India, nearly all women reported that ‘most of the time’ family members including husbands and in-laws, encouraged their alcohol use during pregnancy [54].

Early pregnancy was a particularly challenging time for women who wished to conceal their pregnancy status but were in social situations where there were expectations to drink alcohol [29,36,37,39].

*“It’s tough when [the pregnancy] is secret! There is really a social pressure regarding alcohol. It’s crazy!”*

[37]

Receiving support from others played a critical role in influencing women’s capacity to reduce or abstain from alcohol during pregnancy [29,30,33,34,37,38,39,43,44,49]. Women who described feeling supported in environments where abstinence during pregnancy was a norm tended to face fewer barriers to abstention or reduction. Furthermore, for some of these women, alcohol use prior to pregnancy tended to be described as low and sporadic, and therefore did not appear to be integral to their social identity and relationships [37,38,41,42,56].

*“Several women perceived abstinence as a shared norm among their relatives, which made it easier for them to change their alcohol consumption since they did not feel the need to justify themselves”*

[37]

For women lacking social support to reduce or abstain, alcohol reduction or abstinence had a social cost, most commonly experienced through social isolation, judgement, and stigma for women’s personal choices [28,45,47,49,51]. In one study, participants described how their social and familial role had changed since they stopped binge drinking or drinking during pregnancy.

*“I’m a major outcast because I don’t drink, I don’t smoke, I don’t do the drugs. When my grandparents have birthdays and stuff, they don’t invite me.... they think I’m high-class. Any person who is pregnant, they become a designated driver… You become an adult babysitter”*

[45]

Eleven articles explored the role of partners in influencing women’s continued use, reduction, or abstention from alcohol during pregnancy [30,33,34,37,38,43,47,49,54,55,56]. In more supportive contexts, partner’s support was most commonly expressed through: shared beliefs about women’s alcohol abstinence during pregnancy [30,38,44]; joint alcohol use decisions [30,38,55]; support to resist temptations to use [33,34,37]; partner’s reduction or abstinence alongside women [30]; support to conceal early pregnancy status in social settings [37]; and emotional support to prevent relapse among pregnant women with an addiction to alcohol [33,34].

*“Then we stopped drinking… He wanted to stand by me. We actually did it for our baby”*

[49]

Despite some partners’ preference for women to abstain from alcohol use during pregnancy, some women experienced pressure or had their partners’ preferences exerted through controlling means, such as monitoring women’s health behaviours, resulting in stress rather than support [38,49]. In these particular cases, it was unclear in the literature if women also wished to reduce or abstain, but rather highlighted how women did not feel self-determining around their decision-making.

Other women experienced a lack of partner support to reduce or abstain, expressed through their partner’s continued drinking [30,37,56].

*“I thought he was gonna be a bit more supportive with having the child, we wouldn’t drink together or he would slow down but, he just carried on as before”*

[56]

A lack of support to reduce or abstain from alcohol was also experienced through partners falsely reassuring women that alcohol use was safe due to inaccurate health information and pressuring them to drink during pregnancy [37,43,47,54].

Other forms of support at the interpersonal level, such as support from healthcare practitioners, largely focused on the nature of information provided to women around alcohol use and pregnancy (see the *Alcohol Use Messaging and Information* theme). Common misinformation provided by healthcare providers about safe(r) alcohol types, such as beer, wine or ciders being cited as less harmful than spirits, or communication around consumption timing during pregnancy informed moderate alcohol norms [29,30,39,40,42,43,45,46,48,50,51,53,54], with low consumption considered a form of abstinence for some [53].

At the population level, commonly held knowledge norms favouring alcohol use in moderation over abstinence during pregnancy shaped women’s social environments and personal relationships with alcohol [30,37,41,42,43,47,48,50,56]. In addition, contributing to moderate alcohol use norms was a pervasive lack of awareness about prenatal alcohol exposure and FASD [29,35,39,40,41,42,45,48,51,54,56], highlighting limited public knowledge of the risks associated with alcohol use during pregnancy. While institutional level practice, policy, and knowledge norms concerning alcohol risks and FASD may illuminate further contextual barriers informing this situation, they were not identified in the included literature.

### 4.2. Stigma

Discriminatory views about women who consume alcohol during pregnancy were commonly held and experienced by women themselves at the interpersonal level [30,37,39,42,44,49,51,53]. These were most evident among women in environments where alcohol abstinence during pregnancy was viewed as a simple task that informed social norms and moralistic ideals regarding motherhood.

*“If they drink, they don’t deserve to have a baby. I’m sorry, but they don’t. Because they’re not thinking of the baby. They’re thinking of themselves”*

[44]

Connected to this, women in several studies sought to distance themselves from stigmatizing perceptions of ‘bad’ mothering, aligning themselves instead with the dichotomizing and stigmatizing construction of ‘good’ or ‘responsible’ mothering [38,42,44,49,50,56].

For some women, fear of judgement formed a key motivator to reduce or abstain from alcohol [39,42], while for others, it caused alcohol use to become hidden, impacting their social connectedness [51].

*“I’ve sort of become more aware of … how I look, so you sort of don’t feel as comfortable, I guess. Even though you might drink at home, in public you sort of feel a bit scrutinised sometimes. People have pretty strong views on it, so for me, I’ve tended to go out less to have a drink, whereas I might have a drink at home”*

[30]

Fear of judgement from healthcare practitioners also contributed to hidden alcohol use for some women, irrespective of the quantity of alcohol consumed [33,34,35,40].

*“I suppose being pregnant you don’t intentionally want to harm your baby. I know a lot of friends who still drink small amounts while they’re pregnant but I don’t know whether truthfully if they were asked whether they drink what they would say, I suppose there’s those barriers, whether people think they can be honest with those sorts of things”*

[35]

Some women who could not abstain from alcohol during pregnancy, however, also feared punitive consequences from potential child welfare or justice involvement [33,34]. While exploration of internalized stigma was not highly prevalent in this literature, two studies discussed women’s shame and guilt for alcohol use and an associated lack of confidence in their personal capacity to parent [28,49].

### 4.3. Trauma and Other Stressors

Pregnancy was met with varying degrees of stress dependent on women’s life circumstances. While women who used alcohol while pregnant differed in their consumption levels, at an individual level, alcohol commonly played an important role in aiding relaxation and for managing or coping with trauma and stress [28,30,33,34,36,41,43,48,49,51,52,54]. As such, for women experiencing stressful circumstances, the benefits of drinking alcohol often outweighed the risks of PAE.

*“I just know that it gives me just that total relaxation feeling… which I guess could outweigh the fact that you’re having alcohol”*

[48]

Moreover, women experiencing stressful circumstances tended to experience multiple barriers to reducing or abstaining from alcohol use during pregnancy, rather than simply one or two challenges [28,45,49,51].

*“I would have gotten more stressed out if I hadn’t drunk during pregnancy. It would have been harder”*

[51]

Only six of the included studies [28,33,34,45,46,49] explored the nature of women’s stressors including addiction; lack of access to essential resources, including healthy food, housing, and income; unstable or unsupportive relationships; domestic or intimate partner violence; and a lack of family and peer support. Women dealing with alcohol and other substance addictions often faced complex barriers and emotional upheaval related to navigating addiction and risks of personal and fetal harm.

*“I was very scared. I was afraid my parents would ask me to leave the house. I was thinking how my first born was given to my parents by the social workers. I didn’t have an income and my husband did not support me in any way. I panicked all the time because I did not know where I was going to live with this baby”*

[28]

Two studies found that for women experiencing multiple stressors, often there was a lack of connection to their baby, which was compounded among women with unwanted pregnancies [28,49]. Further, women’s low-trust, anger, and previous experiences of trauma associated with healthcare practitioners, created barriers to women’s care [33,34].

*“The mothers’ own low level of trust in people, combined with what they perceived as lack of understanding from providers, sometimes caused women to express anger at providers, withdraw from traditional care, continue care tentatively, or minimize contact with physicians and nurses”*

[33]

### 4.4. Alcohol Use Messaging and Information

In the included studies, a lack of public awareness about risks of alcohol use during pregnancy was exacerbated through women receiving abstinence messaging and limited provision of brief intervention and support, screening, or follow-up from healthcare practitioners [37,40,48,51,52,53,55].

Connected to this, in nine studies women reported that their healthcare practitioner had endorsed alcohol use during pregnancy for various reasons, including: for relaxation [46], satisfying occasional alcohol cravings [30], for cardiovascular health [54], the building of blood during pregnancy [44] and for the benefit of the baby [30]. The health professionals considered low levels of alcohol use as safe and downplayed associated risks [39,42,43,50,55].

*“My midwife said that having a glass of red wine was actually better for the baby”*

[30]

Practitioner endorsements evidently impacted women’s alcohol risk awareness and alcohol use choices during pregnancy. Such endorsements from professionals appear to reflect the lack of scientific consensus about low alcohol use risks and changing consumption guidelines over the past decade [29,30,35,36,37,41,47,48]. Gibson et al. (2020) described this by saying,

*“Many non-Indigenous women were aware that the research evidence for harm associated with low or occasional alcohol use was inconsistent and often described low level drinking as being safe.”*

Furthermore, conflicting advice and evidence from women’s family members and social networks increased confusion and, in some cases, affirmed women’s choices to continue drinking [29,30,41,43,48,50,53].

*“The problem is that we hear a bit of everything. … We learn a little bit of information everywhere, and we say, ‘All right, let’s split the difference. We diminish, or we drink a sip, and that’s all’”*

[37]

Conflicting advice created confusion and stress for some women when trying to decipher what was safe during pregnancy, limiting their capacity to make informed choices. For some women, this confusion prompted moderation [37,40,50], while others erred on the side of caution, choosing abstinence [37,41,48]. Furthermore, the framing of alcohol messaging as abstinence-only resulted in women feeling the advice was exaggerated, unconvincing or even controlling [41,45].

*“Some of them [billboard messages promoting abstinence during pregnancy] exaggerate a little bit more than what it should be. Where they have the baby drinking the 40 oz …even though the baby is drinking with it, the baby isn’t going to sit there and turn no 40-oz up to its mouth. That is overexaggerating”*

[45]

Furthermore, the single-focused messaging of abstinence fostered stigma towards women who consume any amount of alcohol during pregnancy, particularly among those who for a multiplicity of complex reasons were unable to reduce or abstain [28,41] and those that did not see their own socioeconomic realities reflected in messaging or advertising [45,49].

*“…if you have any alcohol at all, you’re a bad person, you’re harming your unborn child, you don’t care, that’s the message that’s coming out; a very judgmental, a very policing, that kind of message”*

[41]

No studies included consideration of the role of harm reducing, trauma-informed, or non-stigmatizing messaging. However, some mention was made by women that being adequately informed about alcohol use risks and abstinence guidelines could facilitate reduction or abstinence [35,39].

*“Women also reported that advice from health professionals was a factor that strongly influenced their choices and behavior during pregnancy. Hence, another positive motivation was to comply with professional advice. For those who had received advice to abstain, this strengthened their decision to avoid alcohol during pregnancy”*

[39]

Regardless of messaging, women who directly or indirectly knew someone with FASD were more aware of the types of challenges that individuals with FASD and their families could face on a daily basis, and consequently, strongly mediated their acceptance of abstinence guidelines [29,41,45,47,51].

*“I guess my mom works with children with FASD, so I understand what happens when you drink during pregnancy. But I also think that there are people out there, that probably don’t understand the risks”*

[29]

### 4.5. Access to Trusted, Equitable Care, and Essential Resources

Despite the importance of women accessing prenatal health services during pregnancy, there was a limited focus on how women can access trusted and equitable care. In two studies, women in recovery from alcohol and other substance addiction stressed the importance of non-judgmental approaches and healthcare practitioners being compassionate towards their circumstances [33,34].

*“All the participants suggested ways to improve health care for recovering women. These suggestions included, ‘understand their situation,’ ‘be there for them,’ and ‘be gentle’”*

[33]

In another article by Hocking, O’Callaghan and Reid (2019), one participant reflected on the importance of women receiving quality prenatal care that has continuity and is individually tailored to women’s needs [40].

*“… it could be good to have a couple of familiar faces, that’s when you build the kind of relationship where you feel comfortable talking in-depth. and asking questions. And maybe have a bit more time to explain, so you can ask, ‘Hey remember last time when I had this question about this, can we follow it up?’”*

[40]

## 5. Discussion and Application of the Stigma Action Framework

This systematic review described and synthesized the contexts, conditions and factors influencing women’s use of alcohol during pregnancy identified from the available literature. Throughout the included studies, pregnant and recently postpartum women articulated the duality of alcohol being a component of their social environments and experiencing varying degrees of social pressure to drink [29,36,37,39], while simultaneously experiencing or holding their own discriminatory views around alcohol use during pregnancy [30,37,39,42,44,49,51,53]. Women also expressed the challenges in accessing reliable information about alcohol use in pregnancy, with many being confused after receiving inconsistent or contradictory messaging and a lack of consensus around safe levels of alcohol use during pregnancy [29,30,35,36,37,41,47,48]. Other women, who received abstinence-only information, articulated that this approach felt controlling and increased stigma, particularly when they were unable to reduce or abstain from alcohol use [28,41]. These feelings were exacerbated among women who felt unable to discuss alcohol use with their healthcare providers out of fear of judgement, child removal, or criminalization [33,34,35,40]. The associated guilt and shame can impact women’s ability to access care or their confidence to parent [28,49,57].

These dualities are fundamental to understanding the complexities of alcohol and other substance use during pregnancy. By using the *Stigma Action Framework* to explore influencers across different levels, the results highlighted how, despite the literature’s focus on individual choice and prenatal alcohol use, the barriers and facilitators to women’s alcohol use were rarely a result of individual choice, but rather a reflection of interpersonal, institutional and population-level factors. Critically, the results also highlight key gaps in this literature where themes were un- and underexplored (e.g., harm reducing policy, practice, and alcohol use messaging; the role of safe, trusted; and accessible services in supporting women during pregnancy and postpartum periods). In Table 3, we demonstrate the findings of this review across the themes and different levels, while highlighting identified gaps in the literature (in grey cells).

We now ground and expand the findings from the systematic review to focus on all four levels of the *Stigma Action Framework*. By doing so, we link individual and interpersonal factors identified by women in the qualitative studies with broader structural factors which are critical to consider in overall health promotion for women, children, and families. In the subsequent sections, un- and underexplored themes are examined and addressed through consideration of how emerging policy and practice approaches described in prevention models in Canada [15,58] and Australia [59], have been used to ameliorate the barriers to reducing or abstaining from alcohol use in pregnancy, as articulated in Table 3. Further, we discuss the capacity of such approaches for supporting women in reducing and abstaining from alcohol use at the individual, interpersonal, institutional, and population levels.

The interventions being applied and evidenced in Canada and Australia include: gender-informed and inclusive awareness building that reaches women, their partners and the public; trauma-, gender-, violence-, and culture-informed and relationship-based brief intervention/support by a range of health and social care providers; access to welcoming, non-judgmental services; and access to services that wrap a wide range of needed practical supports around mothers and their children [16,19,21,32,60,61]. These institutional and population level interventions act as remedies to the challenges cited by pregnant women who use alcohol. In this way, recommendations for action can move beyond the usual recommendations for supporting individual change to be more accurately focused on service and system level changes that have the potential to make individual change possible.

### 5.1. Social Norms, Relationships, and Alcohol Use Information

Despite alcohol use being widely integrated into most women’s social environments and broader population-level social norms, the included studies found that women received varying levels of support and information from their peers, family members, partners, and service providers to reduce or abstain from alcohol use during pregnancy. The literature highlights the influential role of partners, and in several included studies, partners were considered in the research question, [30,37,54,55,56], however, the studies fail to capture the implications of pregnancy, fetal, and infant health being traditionally framed as the sole responsibility of women [38,62,63].

Throughout the findings, women expressed varying levels of partner support. While some studies noted that partners stopped drinking when the pregnancy was confirmed, other partners used the pregnancy period to surveil or police women’s behaviours. Embedding partners into preconception and prenatal care, messaging and support can be important to reducing the burden on women and has the potential to address gendered societal attitudes regarding health promotion and pregnancy and promote important health outcomes for men, women and children [64].

In addition to partners, service providers play an important role in discussing alcohol use and supporting change during pregnancy. However, as the findings demonstrated, practitioner support can be limited [30,39,42,43,45,50,53,55]. Conflicting information from the media and evolving guidelines can influence practitioners to doubt their overall abstinence or alcohol reduction messaging [15,65]. It can result in confusion, mixed messaging, and misinformation among those who wish to know more about alcohol use in pregnancy [29,30,39,40,42,43,45,46,48,50,51,53,54]. Moreover, public awareness campaigns, such as posters, billboards, and warning labels, can further stigmatize women and discourage them from seeking additional supports [66]. The media and public discourse around alcohol use in pregnancy can contribute to harmful narratives about women who use substances and perpetuate misconceptions about women who use alcohol during pregnancy, resulting in providers only discussing substance use with subpopulations that have been stigmatized and falsely stereotyped [16,57].

Throughout the findings, women articulated that they wished to be adequately informed about alcohol use risks and alcohol use guidelines during pregnancy [37,40,48,51] and that informative approaches to discussing alcohol and other substance use facilitated women’s alcohol reduction or abstinence during pregnancy. One strategy that health and social service providers can adopt in order to discuss alcohol and related health issues with women and their partners are brief interventions.

#### Brief Interventions

There are a range of reasons that service providers may not feel confident in discussing substance use with women, including lack of adequate knowledge about current guidelines, fear of jeopardizing their relationships with women, or concerns about being perceived by women as judgmental and stigmatizing [15,67]. However, discussing substance use and how it is connected to other health or social concerns can support women increase their health and wellbeing regardless of their social and structural contexts. In this regard, brief interventions act as collaborative conversations about alcohol and/or other substances and related health issues [15,16,58].

Brief interventions can be conducted by a wider range of health and social service providers (i.e., physicians, nurses, midwives, sexual health service providers, anti-violence workers, Indigenous health workers, etc.) and can include a range of topics such as mental wellness, healthy relationship dynamics, health promotion strategies, and self-care [16,68,69,70]. Moreover, framing brief interventions as ‘doorways to conversation’ may leave room for evidence-informed discussions from a trauma-informed and harm reducing perspective, which may further facilitate women’s alcohol use reduction or abstinence during pregnancy [16].

### 5.2. Access to Trusted, Non-Stigmatizing, and Equitable Prenatal Health and Substance Use Services

Efforts to avoid the harms of intersecting forms of stigma are among the most significant factors deterring women from accessing supportive health care and services [71]. For pregnant women who are aware of the risks of alcohol use in pregnancy but are struggling to abstain because of a multitude of complex factors, judgmental or abstinence-focused responses from health care providers may perpetuate shame and risk isolating women. Women’s isolation can be perpetuated by organizational policies that require women to abstain from substances in order to access services. This can further limit access much needed to substance use, mental health, and harm reduction services, and housing or anti-violence programs [72,73]. Women may avoid accessing prenatal care out of fear of judgement, child apprehension, or criminalization related to their substance use [32,61,71,74,75].

Health and social care providers who lack understanding or training on substance use can have limited understanding of why women may use alcohol in pregnancy and why abstinence may be an unrealistic goal [16,32]. In the studies included in this review, fear of judgement from healthcare providers was noted to contribute to isolation or hidden alcohol use, regardless of alcohol consumption levels [33,34,35,40]. However, the broader implications of stigma on access to trusted and equitable services were narrowly discussed in the included studies, given the strong emphasis on individual [28] or interpersonal [33,35,39,44] experiences of stigma.

The importance of healthcare practitioners’ conveying acceptance, a non-judgmental stance, and an understanding of the contexts of women’s lives and circumstances, is crucial to support access to prenatal care [15,21,33,34,61,76]. Further, women’s preferences for prenatal care continuity that is individually tailored was briefly mentioned in one study [40]. However, the findings did not mention strategies that can help foster women-centered, integrated pregnancy care that can address social and structural inequities.

#### Holistic, Integrated Support for Pregnant Women with Substance Use Concerns

Offering holistic, integrated support for pregnant women with substance use concerns is an emerging best practice in how to support pregnant women with substance use concerns. These programs can be provided through various models including outreach, multi-service co-located agencies, or a network of community-based services [15]. Research in this area has shown that integrated support models can improve maternal and fetal outcomes and successfully support women to reduce alcohol use in pregnancy [15,19,32,61,77].

Using trauma-informed, harm reduction oriented, and women-centered approaches can help overcome the pervasive stigma and other barriers that some women experience when accessing services [61]. Interventions that address social and structural factors (e.g., childcare, housing, transportation, food/nutrition) that affect women’s ability to access services are often more effective in engaging women. For instance, collaborative models where providers work in a multi-service co-located program or network of services can result in higher referrals, attainment of treatment goals, and retained custody and greater reunification with their child(ren) if custody was lost [78,79]. Co-location of service providers can also create a better stream of communication across services, removing barriers and increasing women’s access to services [80]. Moreover, as these programs offer comprehensive, wraparound services for pregnant and parenting women with substance use concerns, they are developed to match community needs and resources [81]. In Canada, researchers have evaluated both integrated substance use treatment programs in Ontario [20,74,77,81] as well as multi-service community-based FASD prevention programs [32,61].

Many women accessing these comprehensive services addressed their desire to quit or reduce substance use upon learning they were pregnant, but had experienced violence and trauma that was linked to mental health and substance use concerns [32]. These programs offer women support related to substance use and/or trauma, but also with child welfare support, fostering of mother-child relationships, and information related to pregnancy [61]. Women noted that what they liked most about their program was staff and their non-judgmental, supportive, and helpful approach; the friendships and sense of community they developed; the safe and healthy environment; and the ability to access multiple services that could positively impact them and their children [19,32,61].

Other forms of services, such as home visitation and case management models, can empower women to make healthy lifestyle changes through holistic, trauma-informed, and harm reduction-oriented care [82]. These models can also decrease substance-exposed pregnancies by linking pregnant women and mothers to community resources that will help them build and maintain healthy, independent family lives and aid in reducing stressors and connected isolation that may drive alcohol use [83,84]. In North America and Australia, the Parent Child Assistance Program (PCAP) has been offered in a range of communities to empower pregnant women and mothers over a three-year mentorship program. PCAP has been adapted to women’s and community’s needs, allowing for cultural adaptation/inclusion, as has been the case in First Nations and Métis communities in British Columbia and Alberta, Canada [21].

Other opportunities for integrated support include training and collaboration across sectors to improve shared understandings of substance use in pregnancy. This helps improve the understanding of the other sector’s role, increases communication and referrals, and strengthens co-operation and partnerships [85]. It can be further reinforced by cross-ministerial collaboration, which can be a crucial bridge in furthering agency and partner collaboration [81].

## 6. Limitations

The current systematic review explored the contextual factors, barriers and facilitators to women’s alcohol use in pregnancy found in qualitative studies. Two studies including women in the preconception period [39,41] did not differentiate their study participants by sub-groups. While this did not preclude the inclusion of these studies as pregnant and recently postpartum women’s voices were discernible, we were not able to confirm the true number of participants described in the current systematic review.

While many of the included articles spoke to societal alcohol use norms and alcohol norms during pregnancy, the largely homogeneous demographic of middle-class, educated women amongst the literature resulted in limited discussion of diverse barriers and facilitators to alcohol use, especially amongst women who are most vulnerable to potentially harmful alcohol consumption during pregnancy. It also negated discussions of inequitable, racially biased, and colonial healthcare policies and practices and other barriers that Indigenous women and women of colour uniquely experience.

## 7. Conclusions

The current systematic review explored the qualitative literature on the barriers and facilitators to alcohol use during pregnancy and in the recent postpartum period. Such attention to women’s perspectives is foundational to improving service responses to pregnant and parenting women who use alcohol. However, many of the included studies focused on individual behavioural approaches to alcohol reduction and abstinence (i.e., focus on women’s personal views, behaviours, and motivations) with little attention to exploring the potential contextual influences on alcohol use (i.e., structural determinants of health).

The use of the *Stigma Action Framework* as an analytic framework allowed for building upon the findings from qualitative studies, to include an understanding of the contexts and social and structural determinants that impact women’s alcohol use and access to care. Using this broader analytic framework was key to expanding the focus away from individual change approaches towards those that address social and structural factors in shaping women’s use of alcohol, service access and empowerment.

Designing service systems that address needed changes identified by women, such as a lack of positive involvement of partners, lack of clear and consistent messaging around the risks of alcohol use in pregnancy, fear of judgement from service providers, fear of being further stigmatized, personal and social pressures to consume alcohol-can best be realized when the pervasive influences of structural determinants of health are understood and addressed.

Advances towards reducing barriers and facilitating support are being applied and documented in countries like Canada and Australia. Key to supporting change in these countries has been accessible services that include co-located supports and services for health, housing, parenting, nutrition, substance use, family violence and related trauma, and other practical supports. Foundational to these approaches are philosophies based on respect, self-determination, reducing harms, preventing re-traumatization, and supporting relational connections among women, their peers and service providers. These approaches elevate the findings from this review to promote organizational and systemic change that remove the burden from women’s shoulders and expand the responsibility for change in alcohol use in pregnancy to also include health and social service organizations, policy makers, funders, and society as a whole.

## Figures and Tables

**Table 1:** Stigma Action Framework.

	Level of Stigma	How Stigma is Operationalized
Individual	Person who experiences stigma	Unfair treatment Internalized feelings of shame and guilt Anticipated stigma (e.g., may not access support)
Interpersonal	Family, friends, social networks, healthcare and social service providers	Using derogatory or dehumanizing language Intrusive attention and questions Hate crimes and assault
Institutional	Health system organizations, health, community, and social service organizations	Restrictions to care based on behaviours or sociodemographic status Unwelcoming or unsafe environments Institutional policies that cause harm (e.g., low investment of services; unnecessary drug tests)
Population	Mass media, policies, and law	Societal norms and values Widely held stereotypes Discriminatory laws and policies Inadequate legal protection (or lack of enforcement)

**Table 2:** Table of Characteristics.

Author(s) & Year	Country	Method (Orientation, Data Collection, Analysis)	Population(s)	Research Aim	COREQ Critical Appraisal
Barbour (1990) [43]	USA	Semi-structured interviews	20 women in their third trimester of pregnancy	Explore the drinking behaviours and factors that influence alcohol use among pregnant women	Partially fulfilled
Baxter et al. (2004) [44]	USA	Semi-structured interviews; thematic analysis	60 lower-income women who were pregnant or <12 months postpartum and resided in rural Iowa	Identify women’s attitudes, beliefs, and behaviours around alcohol and pregnancy	Partially fulfilled
Bianchini et al. (2020) [52]	Brazil	Semi-structured interviews; thematic content analysis	14 pregnant women who received prenatal care	Investigate the perceptions of the advice pregnant women received from prenatal care providers about alcohol and tobacco use during pregnancy	Inadequately fulfilled
Branco and Kaskutas (2001) [45]	USA	Focus groups; thematic analysis	11 pregnant and recently postpartum Indigenous and Black women	Understand women’s beliefs and opinions regarding alcohol use during pregnancy	Inadequately fulfilled
Brudenell (1996 & 1997) [33,34]	USA	Grounded theory; participant observation, semi-structured interview, diaries; constant comparative analysis	11 women who self-identified as alcoholics/addicts in recovery and were pregnant or recently postpartum (5 pregnant, 6 with infants younger than one year)	Explore women’s concurrent experiences of alcohol and drug use recovery and the transition to parenthood	Partially fulfilled
Crawford-Willams et al. (2016) [30]	Australia	Focus groups; thematic analysis	9 pregnant women and 8 women who were 4–20 weeks postpartum	Identify knowledge gaps about the effects of alcohol use in pregnancy among pregnant and recently postpartum women, and their partners	Partially fulfilled
France et al. (2013) [39]	Australia	Focus group; thematic analysis	23 women who were pregnant, <3 years postpartum, or were considering pregnancy. Mothers and prospective mothers had to have screened positive for alcohol use in the previous month.	Identify effective population-level messaging strategies to prevent prenatal alcohol exposure	Partially fulfilled
Gibson et al. (2020) [29]	Australia	Interviews and focus groups; content analysis	14 Indigenous and 15 non-Indigenous pregnant women aged 18+ years	Explore influences on pregnant women’s alcohol decision-making in a population with frequent and heavy peer drinking	Partially fulfilled
Gouilhers et al. (2019) [37]	Switzerland	Semi-directive joint interviews; thematic analysis	30 couples expecting their first baby in French Switzerland. Couples were not included if mothers did not drink alcohol prior to pregnancy or had an alcohol use disorder	Explore pregnant women and their partner’s experiences of pregnancy related alcohol behaviour change	Partially fulfilled
Grant et al. (2019) [47]	United Kingdom	Visual data production (timelines, collaging, and dyad sandboxes); elicitation interviews; thematic analysis	10 pregnant women who lived in the highest quintile of deprivation (Welsh Index of Multiple Deprivation) and were claiming welfare benefits	Understand pregnant women from low-income communities’ health experiences during pregnancy	Fulfilled
Hammer and Inglin (2014) [50]	Switzerland	Semi-structured interviews; thematic analysis	50 pregnant women experiencing healthy pregnancies in French Switzerland	Identify pregnant women’s perceptions of the risks of alcohol and tobacco use during pregnancy	Inadequately fulfilled
Hammer (2019) [38]	Switzerland	See Gouilhers (2019)	See Gouilhers (2019)	Understand couples’ risk management related to alcohol use during pregnancy	Partially fulfilled
Hocking, O’Callaghan and Reid (2019) [40]	Australia	Phenomenological; semi-structured interview	12 women who attended an initial prenatal appointment within the past two years	Explore and interpret the messages women receive regarding alcohol use during their first prenatal care visit	Partially fulfilled
Holland, McCallum and Walton (2016) [41]	Australia	Semi-structured interviews and focus groups	20 women who were pregnant, recently postpartum, or were planning a pregnancy	Examine pregnant women’s experiences of alcohol consumption and their perspectives on related health advice	Inadequately fulfilled
Jones et al. (2011) [35]	Australia	Semi-structured interviews	12 midwives and 12 pregnant women	Explore midwives’ advice regarding alcohol consumption, how it corresponds to the National Health and Medical Research Council (NHMRC) Low-Risk Drinking Guidelines, and how pregnant women understand and interpret the advice	Partially fulfilled
Jones and Telenta (2012) [36]	Australia	Semi-structured interviews	See Jones et al. (2011)	Explore attitudes around alcohol consumption during pregnancy and factors that may impact women’s ability to follow the recommendations to abstain from alcohol while pregnant	Inadequately fulfilled
Kelly and Ward (2018) [49]	South Africa	Episodic interviews, focus groups; thematic decomposition analysis	14 pregnant or recently postpartum women who were identified as binge drinkers, dependent on, or addicted to alcohol during pregnancy (using the AUDIT screening tool) and were enrolled in the Healthy Mother Healthy Baby programme and 13 community members (4 men, 9 women) ages 18+	Identify social representations of alcohol use among women who drank alcohol while pregnant	Partially fulfilled
Martinelli et al. (2019) [51]	Brazil	Semi-structured interviews; thematic content analysis	14 pregnant women who were identified as at-risk drinkers during pregnancy (using the Brazilian validated revised T-ACE screening tool)	Explore the motivations behind abstinence and alcohol consumption during pregnancy	Partially fulfilled
Meurk et al. (2014) [42]	Australia	Semi-structured interviews; framework analysis	40 pregnant and recently postpartum women, ages 34–39, from the Australia Longitudinal Study on Women’s Health	Contextualize how women understand their personal identity and act upon risk perceptions related to alcohol use during pregnancy	Partially fulfilled
Pati et al. (2018) [54]	India	Structured interviews ^1^, focus groups; thematic analysis	19 women who were lactating in the past three months and reported alcohol consumption during pregnancy, 18 family members, and 20 local community leaders and frontline workers	Explore the beliefs and perceptions of women from the Santal and Munda tribes around alcohol use in pregnancy	Fulfilled
Raymond et al. (2009) [48]	United Kingdom	Semi-structured interviews; thematic analysis	20 pregnant women	Explore pregnant women’s attitudes around alcohol use in pregnancy and towards sources of information about alcohol use in pregnancy following changes in government guidance	Partially fulfilled
Schölin et al. (2017) [56]	United Kingdom, Sweden	Socio-ecological; semi-structured interviews; thematic analysis	21 parents in England and 22 parents in Sweden with an infant <18 months	Examine perceptions and practices of alcohol use during pregnancy in England and Sweden	Partially fulfilled
Sheridan (2018) [46]	USA	Grounded theory; mixed-methods ^2^, survey, semi-structured interviews; content analysis	14 pregnant or parenting girls, ages 13–19 years, enrolled in an alternative high school for pregnant and parenting girls	Explore the experiences and perceptions of substance use, pregnancy, and motherhood among young mothers	Partially fulfilled
Toutain (2010) [53]	France	Online chat rooms; Thematic analysis	42 pregnant women in three Internet chat rooms	Identify future mothers’ perceptions of alcohol consumption during pregnancy through Internet chat rooms	Inadequately fulfilled
Van der Wulp, Hoving and de Vries (2013) [55]	The Netherlands	Focus groups and semi-structured interviews ^3^; content analysis	25 pregnant women and 9 partners	Explore what information pregnant women and their partners receive about alcohol in pregnancy from their partners	Partially fulfilled
Watt et al. (2014) [28]	South Africa	Semi-structured interviews; thematic analysis	12 pregnant and 12 women <12 months postpartum, aged 18+ years	Examine the experiences of pregnant and postpartum South African women who reported alcohol consumption during pregnancy	Partially fulfilled

^1^ For the purpose of this systematic review, only the themes and quotes from the interviews (pertaining to women’s experiences with alcohol in pregnancy) were included; ^2^ For the purpose of the systematic review, only the qualitative data from the interviews was included and analyzed; ^3^ This article contains two studies: one including midwives and the second including women and their partners. For the purpose of this systematic review, only the second study was reported on.

**Table 3:** Overview of the Findings and Gaps in the Literature Across Analytical Themes and Subthemes Related to Barriers and Facilitators to Alcohol Use in Pregnancy.

Themes and Subthemes		Individual	Interpersonal	Institutional	Population
**Social relationships and norms**	***Unsupportive***	Feeling as though there are a lack of alternatives to alcohol use Perception that alcohol use is not risky/harmful	Lack of support from friends, family, and partners to reduce alcohol use Partners unchanged alcohol use Normalized alcohol use in social situations	Abstinence-only policies	Unsupportive norms favouring alcohol use in moderation Misinformation Lack of awareness regarding harms of alcohol use and FASD
	***Supportive***	Personal strengths Feeling connected to the fetus/baby	Support from others to reduce/abstain from alcohol Joint alcohol use decisions with partners	Abstinence-related policies Non-judgmental care	Supportive social norms that normalize alcohol reduction
				Harm reducing institutional policies/culture	
**Stigma** **(as a barrier to reducing alcohol use in pregnancy)**		Limited self-esteem/capacity to seek support Internalized stigma (limiting self-esteem/capacity to seek support)	Judgement related to alcohol use in pregnancy Belief that alcohol use in pregnancy results in an inability to parent	Punitive institutional policies that prompt child welfare or justice involvement	Dichotomous notions of ‘good’ and ‘bad’ mothers
				Discriminatory institutional practices that prejudice based on SES, ethnocultural identity, pregnancy status, alcohol or substance use, or mental health	Discrimination related to SES, gender, mental health status Punitive laws and policies Racism Punitive approaches for alcohol use
**Trauma and Stressors (as barriers to reducing alcohol use in pregnancy)**		Alcohol as a coping mechanism Feeling unsafe Feeling disconnected from the fetus/baby	Lack of trusted relationships/social support network Lack of safety due to another External expressions of trauma Domestic and intimate partner violence	Lack of access to essential resources	Colonial policies Intergenerational trauma Structural disparities (e.g., poverty)
				Lack of outreach/access to care Intergenerational/recent institutional trauma Institutional lack of safety	
**Alcohol messaging and information**	***Harmful***	Confusion around how to interpret information *See internalized stigma and trauma*	Conflicting, unclear and/or harmful messaging from healthcare providers, friends, and family Limited provision of brief interventions and health information related to pregnancy and alcohol use	Abstinence-only, judgmental, and stigmatizing alcohol use messaging, education and policy Gendered care that is only geared towards women’s health	Unclear and evolving national alcohol use policies and guidelines Stigmatizing public alcohol abstinence messages Lack of awareness harms of alcohol use and FASD
					Gendered policies that frame preconception and prenatal care as a women’s-only issue
	***Harm Reducing***	*See supportive relationships and norms*	Receiving trusted, clear and consistent messaging from healthcare providers	Trauma-informed, harm reducing, non-stigmatizing messaging and policy Patient-oriented care/information Integration of partners in prenatal care	Harm reduction-oriented policies and guidelines for alcohol use during pregnancy Harm reducing mass media campaigns and messaging
**Access to trusted, equitable care and essential resources (facilitating alcohol reduction/abstinence in pregnancy)**		Access to care without fear of failing to reduce alcohol use	Supportive relationships Support accessing resources Consistent access to prenatal care	Adoption of harm reduction oriented, gender-, violence-, and trauma-informed practice Holistic and integrated pregnancy care Addressing structural disparities Adoption of patient-oriented care Integration of partners in prenatal care	Laws, policies and media supporting women and men’s health and wellbeing Structural security Gender transformative interventions and campaigns for men

Italicized text indicates subthemes. Grey cells indicate where there were gaps in the included literature.

## Supplementary Materials

The following are available online at https://www.mdpi.com/article/10.3390/ijerph18073445/s1, File S1: Summary of Search Terms Used; Table S1: PRISMA 2009 Checklist.
