# Supplementary material for: “The Problem Is that We Hear a Bit of Everything…”: A Qualitative Systematic Review of Factors Associated with Alcohol Use, Reduction, and Abstinence in Pregnancy
Source: Int J Environ Res Public Health. 2021 Mar 26;18(7):3445. doi: 10.3390/ijerph18073445 (PMC8037183; doi:10.3390/ijerph18073445)
Supplement: Supplementary file 1 [file ijerph-18-03445-s001.zip › ijerph-1129780 supplementary/Supplementary Data Files/Supplementary File S1_Database and search terms used.docx]

**Supplementary Data File: Summary of database and search terms used**

**PubMed**

(“Pregnancy trimesters”[mesh] OR "Preconception Care"[Mesh] OR "pregnancy complications"[MeSH Terms] OR "Pregnancy"[Mesh] OR "Pregnant Women"[Mesh] OR "Prenatal Exposure Delayed Effects"[Mesh] OR “fetus”[mesh] OR “in utero”[tiab] OR “Maternal Exposure”[Mesh] OR “Maternal Health Services”[mesh] OR “Prenatal Care”[mesh] OR fetal[tiab] OR Fetus[tiab] OR foetal[tiab] OR foetus[tiab] OR perinatal[tiab] OR Placenta*[tiab] OR Placenta[mesh] OR Pregnancies[tiab] OR pregnancies[tiab] OR pregnancy[tiab] OR pregnant[tiab] OR prenatal[tiab] OR pre-natal[tiab]) AND ("Alcohol Abstinence"[Mesh] OR Drinking behavior[mesh] OR "alcohol drinking"[MeSH Terms] OR "Ethanol"[Mesh] OR “Alcoholic Beverages”[Mesh] OR "Alcohol‐Related Disorders"[MeSH] OR “alcoholism”[MesH] OR alcohol related disorder[TIAB] OR alcohol abuse[TIAB] OR alcohol dependence[TIAB] OR alcohol*[tiab] OR (alcohol[tiab]AND (consumption[tiab] OR consume[tiab] OR drinking[tiab] OR intake[tiab] OR use[tiab] OR stop*[tiab] OR quit*[tiab] OR reduce*[tiab] OR reduct*[tiab] OR “give up” OR “giving up” OR abstinence OR cessation OR exposed[tiab]))) AND (“field work”[TIAB] OR “focus groups”[Mesh] OR "grounded theory"[MeSH Terms] OR ”grounded theory” OR “interviews as topic”[Mesh] OR “key informant”[TIAB] OR “narration”[Mesh] OR “qualitative research”[Mesh] OR “semi-structured” OR ethnograph*[TIAB] OR face-to-face[TIAB] OR fieldwork[TIAB] OR focus group[TIAB] OR focus groups[TIAB] OR guided interview[TIAB] OR hermeneutic[tiab] OR indepth[TIAB] OR in-depth[TIAB] OR informal[TIAB] OR lived experience[tiab] OR mixed methods[tiab] OR phenomenological[tiab] OR qualitative[TIAB] OR semistructured[TIAB] OR structured[TIAB] OR thematic[tiab] OR theme[tiab] OR unstructured[TIAB] OR interview[tiab] OR interviews[tiab] OR themes[tiab] OR interviewing[tiab])

Limits: English , Humans

**Embase**

'pregnancy'/exp OR 'prepregnancy care'/exp OR 'pregnant woman'/exp OR 'prenatal exposure'/exp OR 'fetus'/exp OR “in utero”:ti,ab OR 'maternal exposure'/exp OR 'maternal health service'/exp OR 'pregnancy complication'/exp OR 'prenatal care'/exp OR fetal:ti,ab OR Fetus:ti,ab OR foetal:ti,ab OR foetus:ti,ab OR perinatal:ti,ab OR Placenta*:ti,ab OR 'placenta'/exp OR Pregnancies:ti,ab OR pregnancies:ti,ab OR pregnancy:ti,ab OR pregnant:ti,ab OR prenatal:ti,ab OR pre-natal:ti,ab

'alcohol abstinence'/exp OR 'drinking behavior'/exp OR 'alcohol consumption'/exp OR 'alcohol'/exp OR 'alcoholic beverage'/exp OR 'alcoholism'/exp OR “alcohol related disorder”:ti,ab OR “alcohol abuse”:ti,ab OR “alcohol dependence”:ti,ab OR alcohol*:ti,ab OR (alcohol:ti,ab AND (consumption:ti,ab OR consume:ti,ab OR drinking:ti,ab OR intake:ti,ab OR use:ti,ab OR stop*:ti,ab OR quit*:ti,ab OR reduce*:ti,ab OR reduct*:ti,ab OR “give up” OR “giving up” OR abstinence OR cessation OR exposed:ti,ab))

AND

(“field work”:ti,ab OR “grounded theory” OR 'grounded theory'/exp OR 'interview'/exp OR “key informant”:ti,ab  OR “narration” OR 'qualitative research'/exp OR “semi-structured” OR ethnograph*:ti,ab OR face-to-face:ti,ab OR fieldwork:ti,ab OR “focus group”:ti,ab OR “focus groups”:ti,ab OR “guided interview”:ti,ab  OR hermeneutic*:ti,ab OR indepth:ti,ab OR in-depth:ti,ab OR informal:ti,ab OR lived experience:ti,ab OR mixed methods:ti,ab OR phenomenological:ti,ab OR qualitative:ti,ab OR semistructured:ti,ab OR structured:ti,ab OR thematic:ti,ab OR theme:ti,ab OR unstructured:ti,ab OR interview:ti,ab OR interviews:ti,ab OR themes:ti,ab OR interviewing:ti,ab)

Limits EMBASE, English Human

**CINAHL via EbscoHost**

MH "Prenatal Care" OR MH "Maternal Health Services+" OR MH "Maternal Exposure" OR MH "Fetus+" OR MH "Prenatal Exposure Delayed Effects" OR MH "Expectant Mothers" OR MH "Pregnancy+" OR MH "Pregnancy Complications+" OR MH "Prepregnancy Care" OR MH "Pregnancy Trimesters+" OR MH "Placenta+" OR “in utero” OR fetal OR Fetus OR foetal OR foetus OR perinatal OR Placenta* OR Pregnancies OR pregnancies OR pregnancy OR pregnant OR prenatal OR pre-natal

AND

MH "Drinking Behavior+" OR MH "Alcohol Drinking+" OR (MH "Ethanol+") OR (MH "Alcoholism") OR MH "Alcoholic Beverages+" OR (MH "Alcohol-Related Disorders+") OR OR “alcohol related disorder” OR “alcohol abuse” OR “alcohol dependence” OR alcohol* OR (alcohol AND (consumption OR consume OR drinking OR intake OR use OR stop* OR quit* OR reduce* OR reduct* OR “give up” OR “giving up” OR abstinence OR cessation OR exposed ))

AND

“field work” OR MH "Focus Groups" OR MH "Grounded Theory" OR ”grounded theory” OR
MH "Interviews" OR MH "Semi-Structured Interview" OR “key informant” OR narration OR
MH "Qualitative Studies" OR “semi-structured” OR ethnograph* OR face-to-face OR fieldwork OR focus group OR focus groups OR guided interview   OR hermeneutic OR indepth OR in-depth OR informal OR lived experience OR mixed methods OR phenomenological OR qualitative OR semistructured OR structured OR thematic OR theme OR unstructured OR interview OR interviews OR themes OR interviewing

Limits:English

**PsycINFO** **via APA**

{Pregnancy} OR {Prenatal Exposure} OR {Fetus} OR {Placenta} OR {Prenatal Care}

"Pregnant Women” OR “in utero” OR fetal OR Fetus OR foetal OR foetus OR perinatal OR Placenta* OR OR Pregnancies OR pregnancies OR pregnancy OR pregnant OR prenatal OR pre-natal

AND

{Drinking Behavior} OR {Alcohol Drinking Patterns} OR {Alcohol Drinking Attitudes} OR {Ethanol} OR {Sobriety} OR {Alcoholic Beverages} OR {Alcoholism}

alcohol related disorder OR alcohol abuse OR alcohol dependence OR alcohol* OR (alcohol AND (consumption OR consume OR drinking OR intake OR use OR stop* OR quit* OR reduce* OR reduct* OR “give up” OR “giving up” OR abstinence OR cessation OR exposed))

AND

{Grounded Theory} OR {Interviews} OR {Qualitative Research}

“field work” OR ”grounded theory” OR “key informant” OR narration OR OR “semi-structured” OR ethnograph* OR face-to-face OR fieldwork OR focus group OR focus groups OR guided interview OR hermeneutic OR indepth OR in-depth OR informal OR lived experience OR mixed methods OR phenomenological OR qualitative OR semistructured OR structured OR thematic OR theme OR unstructured OR interview OR interviews OR themes OR interviewing

**Web of Science – Clarivate**

“Pregnancy trimesters” OR "Preconception Care" OR "pregnancy complications" OR "Pregnancy" OR "Pregnant Women" OR "Prenatal Exposure Delayed Effects" OR “in utero” OR “Maternal Exposure” OR “Maternal Health Services” OR “Prenatal Care” OR fetal OR Fetus OR foetal OR foetus OR perinatal OR Placenta* OR Placenta OR Pregnancies OR pregnancies OR pregnancy OR pregnant OR prenatal OR pre-natal

AND

"Alcohol Abstinence" OR “Drinking behavior” OR "alcohol drinking" OR "Ethanol" OR “Alcoholic Beverages” OR "Alcohol‐Related Disorders" OR “alcoholism” OR “alcohol related disorder” OR “alcohol abuse” OR “alcohol dependence” OR alcohol* OR (alcohol NEAR/2 (consumption OR consume OR drinking OR intake OR use OR stop* OR quit* OR reduce* OR reduct* OR “give up” OR “giving up” OR abstinence OR cessation OR exposed ))

AND

“field work” OR “focus groups” OR ”grounded theory” OR “interviews as topic” OR “key informant”   OR “narration” OR “qualitative research” OR “semi-structured” OR ethnograph* OR face-to-face OR fieldwork OR “focus group” OR “focus groups” OR “guided interview” OR hermeneutic OR indepth OR in-depth OR informal OR “lived experience” OR “mixed methods” OR phenomenological OR qualitative OR semistructured OR OR thematic OR theme OR unstructured OR interview OR interviews OR themes OR interviewing
